# Role of Body Composition in Patients with Resectable Pancreatic Cancer

**DOI:** 10.3390/nu16121834

**Published:** 2024-06-11

**Authors:** Annarita Pecchi, Filippo Valoriani, Riccardo Cuoghi Costantini, Denise Squecco, Andrea Spallanzani, Roberto D’Amico, Massimo Dominici, Fabrizio Di Benedetto, Pietro Torricelli, Renata Menozzi

**Affiliations:** 1 Radiology Department, Modena University Hospital, University of Modena and Reggio Emilia, 41124 Modena, Italy; 204556@studenti.unimore.it (D.S.); pietro.torricelli@unimore.it (P.T.); 2 Division of Metabolic Diseases and Clinical Nutrition, Department of Specialistic Medicines, Modena University Hospital, 41124 Modena, Italy; valoriani.filippo@aou.mo.it (F.V.); renata.menozzi@unimore.it (R.M.); 3 Unit of Clinical Statistics, University Hospital of Modena, 41124 Modena, Italy; riccardo.cuoghicostantini@unimore.it (R.C.C.); roberto.damico@unimore.it (R.D.); 4 Oncology Department, Modena University Hospital, University of Modena and Reggio Emilia, 41124 Modena, Italy; spallanzani.andrea@aou.mo.it (A.S.); massimo.dominici@unimore.it (M.D.); 5 Hepato-Bilio-Pancreatic Surgery and Liver Transplantation Unit, Modena University Hospital, University of Modena and Reggio Emilia, 41124 Modena, Italy; fabrizio.dibenedetto@unimore.it

**Keywords:** pancreatic surgery, cancer, CT, body-composition, nutritional status, sarcopenia, adipose tissue

## Abstract

This study investigates the role of body composition parameters in patients with pancreatic cancer undergoing surgical treatment. The research involved 88 patients diagnosed with pancreatic cancer who underwent surgery at the Modena Cancer Center between June 2015 and October 2023. Body composition parameters were obtained from CT scans performed before and after surgery. The percentage of sarcopenic patients at the time of diagnosis of pancreatic cancer is 56.82%. Of the patients who died between the first and second CT evaluated, 58% were sarcopenic, thus confirming the role of sarcopenia on outcome. The study found that all body composition parameters (TAMA, SMI, VFI, and SFI) demonstrated a trend towards reduction between two examinations, indicating an overall depletion in muscle and adipose tissue. We then evaluated the relationships between fat-related parameters (VFI, SFI and VSR) and survival outcomes: overall survival and progression-free survival. Cox univariate regression model show significant parameter related to outcomes was adipose tissue, specifically VFI. The study found that higher VFI levels were associated with greater survival rates. This research holds promise for advancing our understanding of the link between body composition and the prognosis of pancreatic cancer patients.

## 1. Introduction

Despite advances in both diagnostic and therapeutic oncology, pancreatic cancer remains a neoplasm characterized by discouraging survival curves; with five-year survival rates hovering between a mere 5% to 10%, the prognosis for individuals diagnosed with this aggressive disease remains exceptionally challenging [1,2]. Although indeed in recent years there has been an increase in the 5-year survival rate for operated patients up to 17.4%, the survival rate in non-operated patients has remained stable at approximately 0.9% [3]. Analysis of data from recent years has highlighted an increase in both the incidence of new cases and deaths from pancreatic cancer: over the past decade, both the incidence and mortality rates have risen by an average of 0.3% annually [4].

This evidence has positioned exocrine pancreatic cancer as the third leading cause of cancer-related mortality in EU, affecting both men and women [5].

In recent years, researchers have explored various factors contributing to the severity of pancreatic cancer outcomes. One emerging area of interest is the relationship between pancreatic cancer and sarcopenia [6,7,8,9,10,11]. Indeed, preoperative impaired muscle mass has been linked to poorer long-term survival in pancreatic resectable cancer [12,13,14,15,16].

In the literature, a growing number of studies suggest also that body composition represents a relevant factor in cancer patients, capable of influencing the effectiveness of chemotherapy and outcomes in terms of surgical complications, hospitalization times, quality of life and survival [17,18,19,20,21,22,23].

Some data furthermore suggest that adipose tissue loss at diagnosis should be associated with poor survival in pancreatic cancer patients in different clinical setting [24,25].

Similarly recent works highlight that an impaired nutritional status before pancreatic surgery affects many postoperative outcomes: cancer related malnutrition is a recognized risk factor for surgery-related complications [26].

Hence the importance of an accurate analysis of body composition which includes both the quantification of muscle mass and fat mass and the evaluation of their variations over the course of clinical history.

CT and MRI are currently considered the reference standards for body composition analysis, allowing muscle mass and fat mass to be assessed separately [27,28,29].

CT is also performed routinely in the diagnostic-therapeutic process of the cancer patient: at the time of tumor staging, during treatment, to evaluate the response to therapies and in follow-up for surveillance. This method therefore provides an excellent opportunity to integrate the assessment of body composition with the oncological diagnostic data, thus providing essential additional data in the treatment path of the cancer patient.

The CT scan at the L3 level allows to segment the Total Lumbar muscle Area (TLA), which can be analyzed as such or normalized for height (Skeletal Muscle Index: SMI). The same CT scan level consents also to segment visceral and subcutaneous fat area (VFA, SFA), analyzed as such or normalized for height (Visceral Fat Index, Subcutaneus Fat Index) [30,31].

Although the definition of sarcopenia predicts that it comes having primarily documented low muscle strength, numerous authors have equated the presence of low muscle mass and therefore low SMI with the concept of sarcopenia. Many studies have therefore been conducted with the aim of identifying specific cut-offs, or threshold values, of the SMI parameter processed in CT capable of defining the presence of the sarcopenic condition, both in the healthy population and in the sick population.

The relationship between preoperative body composition, muscle and fat parameters, and their impact on overall survival and time to progression in pancreatic cancer remains a subject of uncertainty and debate. Studies on this subject display heterogeneity, and potential biases limit the strength of the conclusions drawn [32,33].

The literature that has focused on the analysis of body composition in patients undergoing surgical treatment of pancreatic cancer is very heterogeneous. Some works have in fact analyzed the correlation between body composition and post-surgical complications [34], others have considered the effect of neoadjuvant chemotherapy on body changes related to surgical outcomes [35]. So some studies include patients undergoing neoadjuvant chemotherapy, others not, others still locally advanced forms resected after chemo-radiotherapy [36] and others patients with pancreatic cancer in various locations and stage [37]. Further dedicated research based on selected and homogeneous populations are needed to provide a definitive understanding of how body composition influences the survival trajectory of pancreatic cancer patients; this is important for refining clinical strategies and improving the comprehensive care of individuals with pancreatic cancer. In light of a recent meta-analysis conducted by Liu et al. [6], it is evident that there is a notable scarcity of studies dedicated to examining body composition specifically within the context of curative-intent surgery, especially when compared to studies focusing on borderline resectable, advanced [37], or metastatic PDAC [38]. Our study, akin to that of Sohal et al. [25], stands out as one of the few to address the role of body composition, especially adipose tissue, in the setting of curative-intent surgery and its impact on survival outcomes. The hypothesis of the work is to explore the role of body composition parameters in the management of patients with pancreatic cancer in order to plan nutritional support interventions that can improve the outcome.

The aim of our work is to evaluate body composition parameters from pre-surgical CT scans, how these parameters change after surgery, the relationship between the various parameters, and their impact on overall survival. Our research holds promise for advancing our understanding of the link between skeletal muscle area, abdominal adipose tissue area, and adipose tissue distribution in patients with pancreatic cancer who underwent surgery.

## 2. Materials and Methods

This retrospective single center study was approved by the local Ethics Committee (Approval number: prot. AOU 0017942/22 ID 3940).

Participants in this study included individuals with histologically proven pancreatic carcinoma candidates to surgical treatment at the University Hospital of Modena between June 2015 and October 2023.

Clinical and nutritional data, including age, gender, height, weight, body mass index (BMI), type of surgery, were collected from medical records and the hospital electronic medical database. The CT images performed by each patient for pancreatic cancer staging before surgery and within six months after surgery were retrospectively reevaluated and the parameters relating to body composition were obtained [30,31].

A GE AW Volume Share 7 workstation, equipped with specialized software, was used.

We selected pre-contrastografic cross sectional images at the third lumbar vertebra (L3) where both transverse processes were clearly identifiable. The software allows the selective visualization of muscle tissues, by establishing threshold values within a density range from −29 to +150 Hounsfield units (HU). This targeted visualization enabled a more accurate segmentation of skeletal muscle tissue.

To quantify these findings, areas of interest (ROI) were delineated using the software tool, aligning with the designated compartments for analysis. Within these ROIs, the software automatically computed the area expressed in square centimeters and the average density value.

**Figure 1:**
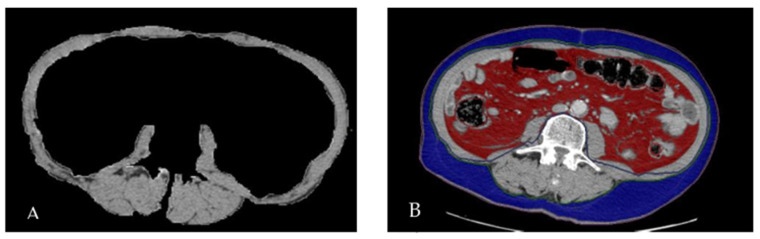
(**A**): TAMA (Total Abdominal Muscle Area) (**B**): VFA (red), SFA (blue) at L3 level.

Total abdominal muscle area (TAMA) (cm^2^) is obtained manually tracing a ROI encompassing the psoas muscles, paraspinal muscles (erector spine, quadratus lumborum, multifidus), and wall muscles (transversus, internal and external oblique, rectus abdominis) at the L3 level (Figure 1A).

The Skeletal Muscle Index (SMI) is cross-sectional TAMA normalized with respect to stature and reported as cm^2^/m^2^; SMI was derived by calculating the ratio between the total area of lumbar muscles and the square of height (cm^2^/m^2^).

IMAC (IntraMuscular Adipose tissue Content): estimate of the adipose infiltration of the muscle, is obtained through the ratio between the average density of the psoas muscle and the density of the subcutaneous adipose tissue.

The body composition parameters of adipose tissue were obtained by establishing threshold values within a typical density range from −180 HU to −30 HU.

Visceral fat area (VFA) (cm^2^): visceral fat area calculated after cutting the subcutaneous fat from the image with selective tissue adipose visceral and subcutaneous (Figure 1B).

VFI (Visceral Fat Index, cm^2^/m^2^): visceral fat area was normalized for height in meters squared and are expressed as cm^2^/m^2^.

Subcutaneous Fat Area (SFA) (cm^2^): subcutaneous fat area was calculated after cutting the visceral fat from the image with selective tissue adipose visceral and subcutaneous (Figure 1B).

SFI (subcutaneous Fat Index, cm^2^/m^2^): subcutaneous Fat Area was normalized for height in meters squared and are expressed as cm^2^/m^2^.

VSR: visceral to subcutaneous adipose tissue area ratios (VSR) to understand abdominal adipose tissue distributions.

Subsequently, patients were divided into sarcopenic and non-sarcopenic groups based on five different predetermined sex-specific SMI cutoff values reported in literature and depicted in Table 1. As highlighted in the review by Amundson et al. [39], numerous studies have been conducted to define and standardize sarcopenia cutoffs. From this review, we extracted several cutoffs that were then analyzed in our study. The cutoffs reported in Table 1 were chosen because they involved the largest and most homogeneous study populations and included a thorough analysis of the study design.

We compared the prevalence of sarcopenic patients before and after surgery using the two different CT examinations. Additionally, we analyzed also the differences and the relationships between all body composition parameters extracted from the two CT scans (TAMA, SMI, IMAC, VFI, SFI).

We also conducted univariate analysis to correlate the body composition parameters with patient outcomes, including progression-free survival, time to progression, and overall survival.

### 2.1. Statistical Analysis

Continuous numerical variables were described using mean and standard deviation, or median and interquartile range (IQR), whereas categorical variables were reported as absolute frequencies and percentages. Comparisons between sarcopenia scores at different time points were performed using McNemar test, while the associations between these scores and other measures of interest (VFI, SMI, SFI, IMAC, TAMA) were evaluated using Student *t* test and Anova test. Association between pre- and post-surgery body composition parameters were analyzed using univariable linear regression model. The results were presented as mean differences (MDs) and they are reported with 95% Confidence intervals (CIs) and *p*-values. Effect size estimated using Cohen’s f-squared statistic were also reported. Effect size has been considered small if f-squared < 0.15, medium if f-squared < 0.35 and large if f-squared ≥ 0.35. Time-to-event analyses were conducted to assess the impact of patients’ characteristics on progression free survival, time to progression and overall survival. For this purpose, univariable Cox Proportional Hazard models were estimated, and the results were presented as cause-specific hazard ratios (HRs), with 95% CIs and *p*-values. *p*-values below the alpha level 0.05 were considered statistically significant. All analyses were carried out using R version 4.3.2 statistical software (The R Foundation for Statistical Computing, 2023).

### 2.2. Patients’ Characteristics

The research involved 88 patients diagnosed with pancreatic resectable o borderline resectable cancer who underwent surgery with curative intent at the Modena Cancer Center between June 2015 and October 2023. The average age of patients at diagnosis is 75 years, with 51% being male. All patients included in the study underwent surgery, with 56% undergoing pancreaticoduodenectomy, 35% undergoing total pancreatectomy, and 9% undergoing another type of surgery (Table 2).

The pre-surgical staging CT scan of all 88 patients was analyzed to calculate body composition parameters at baseline. Of the 88 patients, only 64 had CT available at six months for comparison of post-surgery body composition parameters; 12 patients died before six months and 12 dropped out of follow-up from our center.

### 2.3. Body Composition Analysis

As expected, the application of different threshold values produced different percentages of sarcopenic patients in our population (Table 3).

The assessment of these threshold values in terms of adequacy with our population was conducted by considering their variability between pre-surgery and post-surgery using the McNemar test. The threshold value that exhibited the most significant differences between the two time points was the one proposed by Prado (*p*-value: 0.052). Before surgery, as shown in Table 3, the threshold suggested by Prado had the highest proportion of sarcopenic patients (56.82%). However, during the follow-up CT scan within six months after the intervention, the same threshold showed a lower sarcopenic rate of 37.50%. This discrepancy can be attributed to 12 patients exiting the follow-up before the six-month CT scan, and 12 patients dying shortly after the surgery due to complications. Among these deceased patients, 7 (58%) had been classified as sarcopenic at the baseline CT scan, contributing to the overall reduction in the number of sarcopenic patients according to Prado.

It also implies that, unlike the other thresholds, the one proposed by Prado highlights a higher percentage of deaths in sarcopenic patients immediately following the intervention (14%) compared to other thresholds such as Ryu (11.6%), Okumura (9.6%), and Ninomiya (12.5%). The threshold proposed by Sui is excluded due to the very small sample size.

Subsequently we conducted a descriptive analysis of all body parameters obtainable from pre and post-surgery CT scans, as presented in Table 4.

Analyzing the average value of the body composition parameters between pre and post CT, it is observed that all parameters (TAMA; SMI; VFI and SFI) show a tendency to reduction, highlighting an overall depletion of muscle and adipose behavior which is more evident for visceral fat and subcutaneous.

The relationships between the various body composition parameters (SMI, TAMA VFI, SFI and IMAC) were explored using univariate linear regression models. These analyses reveled that VFI and TAMA or SMI are interrelated and exhibit direct proportionality. This suggests that when one parameter decreased, the other tend to decrease as well (Table 5). In addition, the associated effect sizes were medium (f-squared between 0.15 and 0.35), indicating that TAMA and SMI were relevant factors to describe VFI variability.

### 2.4. Impacts of Body Composition on Recurrence and Mortality in Patients with Pancreatic Cancer

Later, through the Cox univariate regression model, we found that the statistically significant parameter in relation to outcomes was not sarcopenia but rather body adipose tissue, specifically VFI (HR 0.98; 95% CI, 0.97–0.99; *p* = 0.003).

Subsequently, we carried out an in-depth univariate analysis of VFI, SFI, and VSR concerning overall survival, time to progression, progression-free survival. The data pertaining to time to progression and progression-free survival are overlapping, so only progression-free survival will be taken into consideration.

We performed exploratory analyses to establish the optimal cut-off values of VFI and SFI and VSR to distinguish patients with poor prognostic body composition. Using the maximally selected rank statistics, the cut-off values for VFI were 50 cm^2^/m^2^, those for SFI were 50 cm^2^/m^2^, and those for VSR were 1.00. In our sample, eight patients were found to have a VFI greater than 100. Due to the small number and for the sake of standardization, these patients were grouped into the category of “over 50”. This procedure was adopted to ensure that the results of the analysis are representative and reliable, considering the limited number of patients with a VFI greater than 100 in the sample.

**Figure 2:**
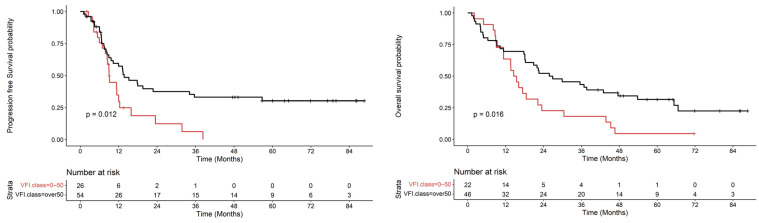
Time to progression and Overall Survival curves according to VFI. The red color indicates the class that includes VFI values between 0 and 50. The black color includes VFI values greater than 50.

As illustrated in Figure 2, there is a notable reduction in progression free survival (*p* = 0.016) and overall survival (*p* = 0.012) for VFI values less than 50 cm^2^/m^2^. These results imply that patients with higher VFI level exhibit greater survival rates compared to the low VFI group in pancreatic disease.

**Figure 3:**
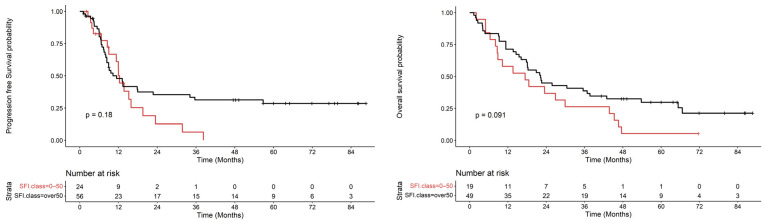
Time to progression and Overall Survival curves according to SFI. The red color indicates the class that includes SFI values between 0 and 50. The black color includes SFI values greater than 50.

SFI showed a similar trend to VFI with respect to survival outcomes (Figure 3) although the correlations did not show statistically significant levels. The data concerning VSR did not produce statistically significant results.

## 3. Discussion

Pancreatic cancer is indeed a deadly disease with an increasing incidence; it represents the third leading cause of cancer death in both men and women in EU, with discouraging five-year survival rates that hovering between a mere 5% to 10% [1,2,4]. To date, surgical resection remains the primary method of achieving a potential cure for PDAC.

However, only a minority of patients are candidates for surgery due to factors such as tumor location, size, involvement of nearby blood vessels, and overall health status. Even among those who undergo surgery, the likelihood of long-term survival is still relatively low.

In recent years, different works in literature underline the impact of individual patient characteristics on survival outcomes, with particular attention given to body composition factors. These factors would have a double role, as predictive factors of survival outcomes and as instrument capable of highlighting unfavorable nutritional metabolic aspects, whose co-correction can allow an improvement in the outcomes themselves. CT is performed routinely in the diagnostic-therapeutic process of the oncological patient: at the time of tumor staging, during treatment, to evaluate the response to therapies and in follow-up. This method therefore provides an excellent opportunity to integrate the assessment of body composition with the oncological diagnostic data, thus providing essential additional data in the treatment path of the cancer patient.

Numerous works have analyzed the impact of body composition factors in patients undergoing surgery for pancreatic cancer, however these studies are quite heterogeneous. Some works analyzed the correlation between body composition and post-surgical complications [34], others have considered the effect of neoadjuvant chemotherapy on body composition [35]. Some studies include patients who have undergone neoadjuvant chemotherapy treatments which still have a significant impact on body composition parameters, others locally advanced forms [36] and others patients with pancreatic cancer in various locations and stage [37]. Our study has the advantage of a selected homogeneous population of patients in which the assessment of body composition parameters was carried out on diagnostic CT before each treatment. All patients also underwent pancreatic resective surgery with curative intent. CT can easily and rapidly provide in a semi-automatic mode numerical parameters representative not only of sarcopenia but also of the visceral and subcutaneous adipose compartment, as well as the quality of the skeletal muscle component. Although the definition of sarcopenia predicts not only reduced muscle mass but also low muscle strength, numerous authors have equated the presence of reduced muscle mass and therefore low SMI with the concept of sarcopenia [40,41]. Indeed, sarcopenia evaluated by CT scores has been linked to poor outcomes following pancreatic surgery.

Unfortunately, the cutoffs for identifying sarcopenia are not well defined, and measurements must be adjusted for height, BMI, and the standardized values must be adjusted based on population characteristics. Many studies have therefore been conducted with the aim of identifying specific cut-offs, or threshold values, of the SMI parameter processed in CT capable of defining the presence of the sarcopenic condition.

We considered how the percentage of sarcopenic patients varied in our population by applying different thresholds reported in the literature in the review by Amundson et al. [39], used in pancreatic cancer, without taking into account the geographical characteristics and population standards. We did this to analyze the impact of the chosen cut-off on our population and to statistically evaluate the best threshold in describing the variability between the two CT exams considered in our population. The best cut off resulting from the statistical analysis was the one used by Prodo et al. and shared by other American authors such as Tan et al., Dalal et al. [42,43]. Therefore, reduced muscle mass was defined using predetermined sex-specific SMI cut-off values: 52.4 cm^2^/m^2^ for men and 38.5 cm^2^/m^2^ for women as Prado et al. reported.

The percentage of sarcopenic patients at the time of diagnosis of pancreatic cancer is 56.82%, confirming literature data [44]. Of the patients who died between the first and second CT evaluated, 58% were sarcopenic, thus confirming the role of sarcopenia on outcome [16,17].

We calculated in two-time CT all muscle (TAMA, SMI) and fat (VFI, SFI and IMAC) related body parameters. The relationships between the various body composition parameters explored using univariate linear regression models shows that VFI and TAMA or SMI are interrelated and exhibit direct proportionality. Moreover, the effect sizes suggested a medium effect of TAMA and SMI in explaining the variability of VFI. In post-surgery TAMA and SMI were also significantly associated to SFI, although, in this case the effect size associated to TAMA was small.

Unlike other studies focused either only on the sarcopenia [33,37,45,46] or on the sarcopenic obesity [18,19], our study also evaluated the relationships between the muscular and adipose compartments, analyzing all the quantitative and qualitative parameters of the muscle (TAMA, SMI and IMAC) and both visceral (VFI) and subcutaneous (SFI) fat and our results underline how in pancreatic cancer the muscle composition parameters are closely related to those of the adipose compartment.

However, the correlation between IMAC and muscle parameters was not significant both pre and post-surgery. IMAC, a parameter that reflects muscle quality, is significantly correlated with VFI and SFI at both the pre- and post-surgical CT evaluation, with medium to large effect sizes.

The mean value of all body composition parameters undergoes a reduction in post-surgery imaging. In particular, the greatest depletion is observed in both the subcutaneous and visceral adipose components. This data probably reflects the impoverishment and state of malnutrition commonly found in patients with pancreatic cancer caused not only by reduced food intake but also by malabsorption due to exocrine insufficiency or by pro-inflammatory state established. Surgery can contribute to accentuating the condition of malnutrition [47]. This result also highlights a strong correlation in pancreatic cancer between the body composition parameters of the two compartments: muscle and adipose tissue.

Cox univariate regression model show that the significant parameter related to outcomes was not sarcopenia but rather body adipose tissue, specifically VFI. We then evaluated the relationships between fat-related parameters (VFI, SFI and VSR) and survival outcomes: overall survival and progression-free survival.

Our results demonstrated that there is a notable reduction in progression free survival (*p* = 0.016) and overall survival (*p* = 0.012) for VFI values less than 50 cm^2^/m^2^. VFI is a parameter that reflect the amount of fat in the visceral compartment corrected for individual factors such as height. This baseline parameter was significantly correlated with disease-free survival: when the VFI value is low or lower than the statistically established cut-off, disease-free survival is shorter.

These results imply that patients with higher VFI level exhibit greater survival rates compared to the low VFI group in pancreatic disease. Similarly, the same relationship was demonstrated between SFI and disease-free survival although with lower levels of significance.

The close correlation between VFI and SFI with time to progression can in some way be explained if we consider both parameters representative of the nutritional state and, when reduced, a possible expression of the condition of malnutrition frequently detected in this neoplasm. In this way it is possible to deduce that patients with greater depletion of the adipose component at the onset of the disease may be in a condition of greater malnutrition which significantly negatively impacts time to progression.

Our results are consistent with several previous studies that demonstrated a significant correlation between higher visceral adipose tissue (VAT) loss and worse survival in patients with resectable or unresectable pancreatic cancer [24,48,49]. As reported in these studies detrimental effects of adipose depletion on patient outcomes in cancer may be due to potential multifactorial mechanisms including the action of pro-inflammatory cytokines [24]. In general, the depletion of the adipose compartment at diagnosis or during the disease can be considered a negative prognostic factor. Different authors conclude that multiple therapeutic strategies against involuntary loss of adipose tissue and skeletal muscle mass need to be established to improve the prognose of patients with pancreatic cancer [26,42].

VSR, however, did not give significant results with respect to outcomes and this can be explained by considering this parameter indicative of the relationship between the two adipose compartments and of visceral adiposity in general. In other works, visceral adiposity or the prevalence of the visceral compartment over the subcutaneous one has been negatively correlated with survival outcomes in a possible relationship with the pro-inflammatory role of this adipose component. In our study the correlations of VSR with outcome were not significant.

In conclusion, the VFI and SFI parameters calculated at the onset of the disease can be considered indicators of the time to progression in pancreatic cancer undergoing surgery in association with the evidence of a high incidence of sarcopenia in this population. This strengthens the evidence of the role of nutritional and physical support interventions on pancreatic cancer patients, aimed at correcting malnutrition and increasing the muscular component. In this context, our results confirmed the importance of a Nutritional Oncology Board (NOB) in daily clinical practice, for the multidisciplinary assessment of cancer patients and their early nutritional support from diagnosis [24].

As reported in Sandini et al. [50], nutritional support during neoadjuvant treatment aimed at maintaining and strengthening the muscular component has advantageous results as it is independently associated with a greater rate of surgical resectability. This underlines the role of body composition in identifying factors which, once corrected, can act positively on outcomes.

Further confirmatory studies are necessary to highlight the change in body parameters in relation to nutritional interventions and the impact on outcomes.

We acknowledge some limitations in our study. Firstly, our research was retro-spective and conducted within a single institution, resulting in a small sample size. Moreover a portion of patients was lost to follow-up, not allowing us to have a second temporal evaluation numerically comparable to the first group. However we considered a homogeneous population with resectable or borderline resectable pancreatic cancer. To validate our findings, larger prospective studies are necessary.

## 4. Conclusions

Our results highlight a strong correlation between muscle wasting and fat depletion as result of high catabolic stress related to pancreatic cancer. We confirm the role of CT not only as an excellent tool for diagnosis and monitoring of oncological diseases but also instrument to providing body composition parameters which can be integrated into the evaluation of the patient’s nutritional conditions and used as indicators of outcomes. In particular, in our study, the depletion of the visceral adipose component at diagnosis was significantly correlated with the time to progression.

Alongside tumor-specific prognostic factors, assessing body composition variables before surgery could aid in risk stratification and guide clinical decisions for pancreatic cancer patients. Furthermore, the early identification of metabolic situations related to reduced outcomes allows the implementation of nutritional and physical activity strategies capable of correcting suboptimal body composition parameters.

## Figures and Tables

**Table 1:** Definition of sarcopenia according to different SMI cutoffs reported in the review by Amundson et al. [39].

Author	Year	Study Population	n	Method	Sarcopenia Cut-Off
Ninomiya	2017	Japan	265	Retrospective	M < 43.75 cm^2^/m^2^ F < 38.5 cm^2^/m^2^
Okumura	2017	Japan	302	Retrospective	M < 47.1 cm^2^/m^2^ F < 36.6 cm^2^/m^2^
Sui	2018	Japan	335	Retrospective	M < 40.5 cm^2^/m^2^ F < 33.5 cm^2^/m^2^
Prado	2008	Canada	250	Retrospective	M < 52.4 cm^2^/m^2^ F < 38.5 cm^2^/m^2^
Ryu	2020	South Korea	548	Retrospective	M < 50.18 cm^2^/m^2^ F < 38.63 cm^2^/m^2^

**Table 2:** Numeric variables and descriptive statistics of the studied population.

Categorical Variable	Frequency		Percentage (%)
Gender			
Male	45		51.14
Female	43		48.86
Surgery			
Pancreaticoduodenectomy	48		54.55
Pancreatectomy	31		35.23
Other	9		10.23
**Numeric Variable**	**N**	**Mean**	**Standard Deviation**
Age	88	74.60	10.26
Height (m)	88	1.65	0.10
BMI pre- surgery	38	24.78	3.37

**Table 3:** Sarcopenic patients according to different thresholds before and after surgery.

	Frequency	Percentage (%)	Comparison with Pre-Surgery (*p*-Value)
Sarcopenia Pre-Surgery			
Ryu	43	48.86	-
Prado	50	56.82	-
Okumura	31	35.23	-
Ninomiya	24	27.27	-
Sui	6	6.82	-
Sarcopenia post-surgery			
Ryu	25	39.06	0.453
Prado	24	37.5	0.052
Okumura	17	26.56	0.146
Ninomiya	24	37.5	0.228
Sui	8	12.5	0.579

**Table 4:** Data related to body composition obtained from CT scans before and after surgery.

Variable	N	Mean	Standard Deviation
Pre-Surgery			
TAMA	88	125.31	27.93
SMI	88	45.4	7.33
IMAC	79	−0.27	0.19
VFI	88	63.26	30.5
SFI	88	68.68	35.72
Post-surgery			
TAMA	64	114.24	26.5
SMI	64	42.19	8.08
IMAC	32	−0.37	0.63
VFI	64	42.03	28.35
SFI	64	49.39	35.85

**Table 5:** Association between pre- and post-surgery body composition parameters.

Variable	MD	95% CI	*p*-Value	Effect Size (f^2^)	Effect Size (Class)	Outcome
Pre-Surgery						
TAMA	0.417	(0.203, 0.630)	<0.001	0.170	Medium	VFI
SMI	2.051	(1.286, 2.816)	<0.001	0.321	Medium	VFI
IMAC	73.847	(39.804, 107.891)	<0.001	0.235	Medium	VFI
SFI	0.385	(0.224, 0.546)	<0.001	0.255	Medium	VFI
TAMA	−0.028	(−0.299, 0.242)	0.838	0.000	Small	SFI
SMI	0.822	(−0.193, 1.837)	0.116	0.029	Small	SFI
IMAC	111.055	(76.248, 145.863)	<0.001	0.508	Large	SFI
VFI	0.528	(0.307, 0.749)	<0.001	0.255	Medium	SFI
TAMA	−0.001	(−0.002, 0.001)	0.432	0.008	Small	IMAC
SMI	0.001	(−0.005, 0.007)	0.736	0.001	Small	IMAC
VFI	0.003	(0.001, 0.004)	<0.001	0.235	Medium	IMAC
SFI	0.003	(0.002, 0.004)	<0.001	0.508	Large	IMAC
Post-surgery						
TAMA Post	0.361	(0.110, 0.611)	0.006	0.128	Medium	VFI Post
SMI Post	1.591	(0.813, 2.369)	<0.001	0.259	Medium	VFI Post
IMAC Post	21.863	(5.620, 38.106)	0.013	0.232	Medium	VFI Post
SFI Post	0.594	(0.464, 0.724)	<0.001	1.295	Large	VFI Post
TAMA Post	0.383	(0.060, 0.706)	0.023	0.087	Small	SFI Post
SMI Post	1.741	(0.725, 2.756)	0.001	0.182	Medium	SFI Post
IMAC Post	25.342	(5.058, 45.626)	0.020	0.200	Medium	SFI Post
VFI Post	0.950	(0.742, 1.158)	<0.001	1.295	Large	SFI Post
TAMA Post	0.002	(−0.006, 0.010)	0.614	0.009	Small	IMAC Post
SMI Post	0.021	(−0.006, 0.049)	0.144	0.075	Small	IMAC Post
IMAC Post	0.009	(0.002, 0.015)	0.013	0.232	Medium	IMAC Post
SFI Post	0.007	(0.001, 0.012)	0.020	0.200	Medium	IMAC Post

## Data Availability

Data are available on request from the authors.
